# Radiographic assessment of the correlation between maxillary sinus dimensions and greater palatine canal pathway in CBCT images (a retrospective study)

**DOI:** 10.1186/s12903-025-06782-w

**Published:** 2025-09-16

**Authors:** Dalia Ali Abou-Alnour, Doaa Adel-Khattab, Nermine Ramadan Mahmoud, Marwa EL-Desouky Helal

**Affiliations:** 1 Faculty of dentistry, Modern University for technology and information (MTI), Cairo, Egypt; 2 Oral Medicine, Periodontology and Oral Diagnosis Department, Faculty of Dentistry, Ain Shams University, Cairo, Egypt; 3 Oral Surgery Department, Faculty of Dentistry, October 6 University. 6th of October City, Giza Governorate, Giza, Egypt; 4 Oral Medicine and Periodontology, Faculty of dentistry, Modern University for technology and information (MTI), Cairo, Egypt

**Keywords:** Cone beam computed tomography (CBCT), Reformatted panorama, Greater palatine canal (GPC)

## Abstract

**Objective:**

This study aimed to correlate the dimensions of the maxillary sinus in CBCT-reformatted panoramic radiographs to the greater palatine canal geometry and its neighbor structures in CBCT multiplanar images, so considering the limitations of the current study, we could use panoramic radiographs as an alert tool before performing a variety of implant surgical and dental procedures concerning the Greater Palatine Canal (GPC).

**Methods:**

The GPC pathway and its neighbor structures were assessed in a sample of 48 CBCT Egyptian adult patients’ images (24 females and 24 males) in the three orthogonal planes and correlated to the height, width and hypothetical surface area of the maxillary sinus (MS).

**Results:**

There was a statistically significant difference in sagittal planes among genders regarding GPC pathway, as Type a was the most prominent GPC pathway among females (an antero-inferior direction) in contrast to male cases that shown Type c (posterior-inferior direction, then an anterior-inferior direction) pathway. Male patients had significantly lower GPC angle than female patients (*P* = 0.022). Males had greater mean values of MS height, width & hypothetical surface area than females. The greater the GPC angle in sagittal plan, the lesser MS height, width & hypothetical surface area. In addition, MS of greater height had more posterior extension (depth).

**Conclusion:**

Considering the restrictions of the current retrospective study, the current study illustrated a significant correlation between maxillary sinus (MS) dimensions and greater palatine canal (GPC) anatomy, specifically the GPC pathway and angle. Larger MS (especially height) correlated with GPC with more posterior pathway and lesser angle, which is sex-based association. These findings are with great clinical significance to minimize the risk of GPC neurovascular bundle iatrogenic injury during interventions dealing with posterior maxilla, particularly sinus lifts, implant planning, due to potential impingement from MS expansion.

## Background

Every subfield of dentistry has been developed to provide better surgical and periodontal results, more esthetic and less risk of complications. Precise understanding of the anatomy and pathways of the greater palatine canal (GPC) and greater palatine foramen (GPF) is essential to prevent damage to the greater palatine artery (GPA) during various anesthesiologic, dental, or surgical procedures like dental implant placement. In addition, the pterygopalatine fossa (PPF) can be used to give anesthetic solutions for regional anesthesia and vasoconstrictor solutions to prevent bleeding during endoscopic sinus surgery via the GPC [1–4]. 

Although the anatomy of the GPC has been studied since the mid-1900s [5], a more detailed understanding emerged with the development of the computed tomography (CT) analysis [2, 6, 7]. Although GPC anatomy has been evaluated through many CT studies, the literature is still lacking from ranges of GPC values. Cone-beam computed tomography (CBCT) as an essential modality in dental field was also used to assess the geometric patterns of the GPC and its relation with neighbor structures in the three orthogonal planes [8, 9].

The maxillary sinus (MS) is the earliest to form and the largest paranasal sinus. At 17 weeks, development starts in the womb. It is a crude aerated or fluid-filled slit that lies inferio-medial to the orbit and is oriented longest in the anteroposterior dimension, with a volume range from 60 to 80 mm3 at birth [10]. Over the past 20 years, axial two-dimensional CT, volumetric analysis of three-dimensional scans generated by MDCT and CBCT, and MRI have all been used to assess the development of MS. While the width and length (anteroposterior dimension) of the MS attain adult proportions by the age of 12, the height of the MS rises steadily until the age of 18 [11].

The greater palatine canal s a bony channel located posterior to the maxillary sinus, linking the pterygopalatine fossa to the hard palate through the greater palatine foramen, and offering passage for the greater palatine nerve and vessels [4].

Due to close proximity of the maxillary sinus and the greater palatine canal, it is important to understand their anatomy and precise relation to each other. This understanding helps to prevent or minimize complications during surgical procedures in this area, by avoiding unconscious injury to the neurovascular bundle. Because panoramic radiographs offer low-cost diagnostic tools for maxillary sinus evaluation with a fair availability, while CBCT offers an excellent imaging quality [12–14]. Our study hypothesis was to correlate the dimensions of the maxillary sinus in the CBCT reformatted panoramic radiographs to the greater palatine canal pathways and its neighbor structures in coronal, sagittal and axial CBCT images, so with considering the limitations of the current study we could use 2D panoramic radiographs as an alert before performing a variety of surgical and dental procedures concerning the GPC.

## Methods

### Trial design

This was a retrospective cohort study conducted on 48 CBCT images of Egyptian adult patients (24 males and 24 females to avoid bias in results) obtained from April 2023 to March 2024 and performed by the same operator. As the maxillary sinus and GPC reach its adult size around 18 years old, so patients aged from 18 to 50 years old were randomly retrieved from a database of Planmeca ProMax 3D Mid machine, Helsinki, Finland. CBCT scans were performed with a flat panel detector and a technical parameter of 8 mA, 90 kV, (20*20 cm) FOV, and 13.5 s exposure time, at oral and maxillofacial radiology department, faculty of dentistry. CBCT machine calibration for linear, angular and volumetric measurements, as well as metallic artifact removal is regularly done every 6 to 8 months based on Planmeca recommendations using three phantoms specified for that purpose.

### Study participants

Patients were selected from the out-patient clinic of Department of Oral and Maxillofacial radiology (Faculty of Dentistry, 6th of October). Prior to participation an informed consent written was documented form all participants to ensuring the participants’ comprehension which was approved by the Faculty of Dentistry’s ethical committee at 6th of October (RECO6U/14-2023. The participants were recruited, according to the following inclusion criteria: Patients age were equal to or greater than 18 years old, with high quality maxillofacial CBCT images (well- defined GPC- MS) without any artifacts included in the study. The exclusion criteria were patients who had undergone maxillofacial surgery, any missing or partially disclosed anatomical structures as the first molar tooth, greater palatine foramen (GPF), mid-maxillary suture (MMS), incisive foramen (IF), pterygopalatine fossa (PPF), and neighboring landmarks. CBCT images that showed a partially disclosed maxillary sinus or a partially disclosed greater palatine canal pathway were excluded. CBCT pictures with blatantly unacceptable resolution and artifacts were excluded. Patients with specific syndromes or developmental issues impacting the craniofacial skeleton were excluded. The study was performed by specialists with more than 10 years of experiences.

### Sample size calculation

Calculation of the sample size was done using EPI INFO version 7.2.5.0, according to a study of Howard-Swirzinski, et al. 2010 [4], that reported GPC certain pathway prevalence was (94.5%). The minimal accepted sample size (n) was a total of (80) scanned maxillary sinuses and GPCs, by adopting a confidence interval of (95%), and a margin of error of (5%) with finite population correction.

All of the CBCT scans were assessed using Planmeca Romexis viewer, version 4.6.2R software, where brightness, contrast and sharpness were optimized for visualization of anatomical structures of concern. The following 13 parameters were measured on the reconstructed CBCT sagittal, coronal axial and panoramic views:

Parameters measured in the sagittal planes:

1. The angle formed by the GPC pathway from PPF axis to GPF (Fig. 1a).

**Fig. 1:**
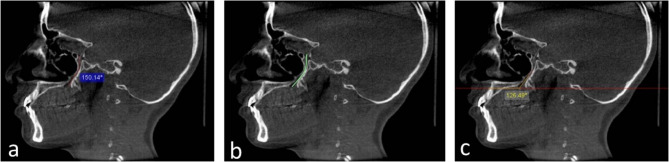
**a** Sagittal CBCT reformatted image showing the angle formed by the GPC pathway from PPF axis to GPF (red curved line). **b** Sagittal CBCT reformatted image showing GPC pathway type (b) (green curved line). **c** Sagittal CBCT reformatted image showing the angle formed between GPC axis and the horizontal plane (yellow curved line)
2. For GPC pathways the following types were developed:
  a GPC travels in an antero-inferior direction through its whole pathway.
  b GPC first travels in an inferior direction, then changes its path into an anterior-inferior direction (Fig. 1b).
  c GPC first travels in a posterior-inferior direction, then then changes its path into an anterior-inferior direction.
  d GPC first travels in a posterior-inferior direction, then in an inferior direction.
  e Other
3. GPC axis-horizontal plane angle (angle formed between a theoretical line perpendicular 146 to the long axis of the body, and the axis of the GPC).

N.B: this angle is drawn through the intersection between a vertical line denoting the GPC axis and a horizontal line drawn through the (draw horizontal line) icon of the used software (Fig. 1c).

The following parameters were measured in the coronal planes:

1. The angle formed by the GPC pathway from PPF to GPF (Fig. 2a).

**Fig. 2:**
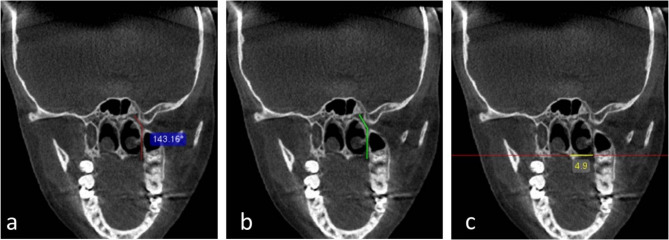
**a** Coronal CBCT reformatted image showing the angle formed by the GPC pathway from PPF axis to GPF (red curved line). **b** coronal CBCT reformatted image showing GPC pathway type (B) (green curved line). **c** Coronal CBCT reformatted image showing GPF-MMS distance (yellow line)
2. For GPC pathways the following types were developed:
  A GPC travels directly in an inferior direction through its whole pathway.
  B GPC travels in an inferior-lateral direction, then directly inferior (Fig. 2b).
  C GPC travels in an inferior-lateral direction, then changes its path into an inferior-medial direction.
  D Other

N.B. where the orientation line run with the long axis if GPC in sagittal plane.

3. GPF-MMS distance in mm (from the center of GPF to the midpoint of the horizontal hard palate, at the level of nasal septum) (Fig. 2c).

N.B: this distance measured by passing the horizontal line available in the software tools through the center of GPF.

The parameters were measured in the axial planes:

1. The distance between the anterior margin of GPF and the distal surface of 1 st molar in mm [GPF(A)−1st molar distance] (Fig. 3a).

**Fig. 3:**
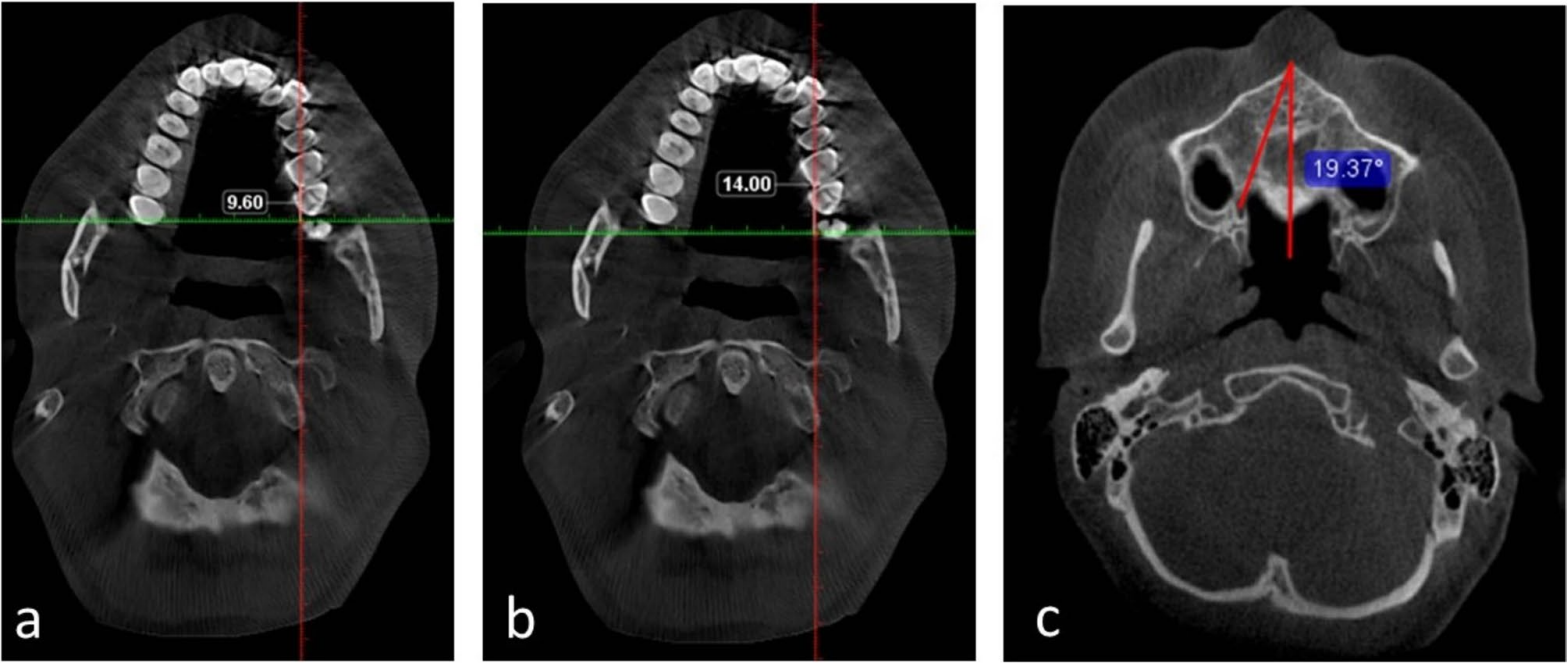
**a** Axial CBCT reformatted image showing the distance between GPF anterior border and the distal surface of 1 st molar (white line). **b** Axial CBCT reformatted image showing the distance between GPF posterior border and the distal surface of 1 st molar (white line). **c** Axial CBCT reformatted image showing MMS–IF–GPF angle (red curved line)
2. The distance between the posterior margin of GPF and the distal surface of 1 st molar in mm [GPF(P)−1st molar distance] (Fig. 3b).

N.B. GPF(A)−1st molar distance and GPF(P)−1st molar distance were measured through passing the horizontal orientation line through the GPF anterior or posterior border, and the distal surface of 1 st molar by the vertical orientation line, where the GPF anterior and the posterior border detection were assured on the sagittal plane side by side with the axial plane.

3 Antero-posterior (AP) dimension of GPF in mm [subtract GPF(A)−1st molar distance from 174 GPF(P)−1st molar distance].
4 Measurement of the MMS–IF–GPF angle (from the center of GPF) (Fig. 3c).

The parameters were measured in the reconstructed CBCT panoramic view:

1. Max. maxillary sinus width in mm (Fig. 4a).

**Fig. 4:**
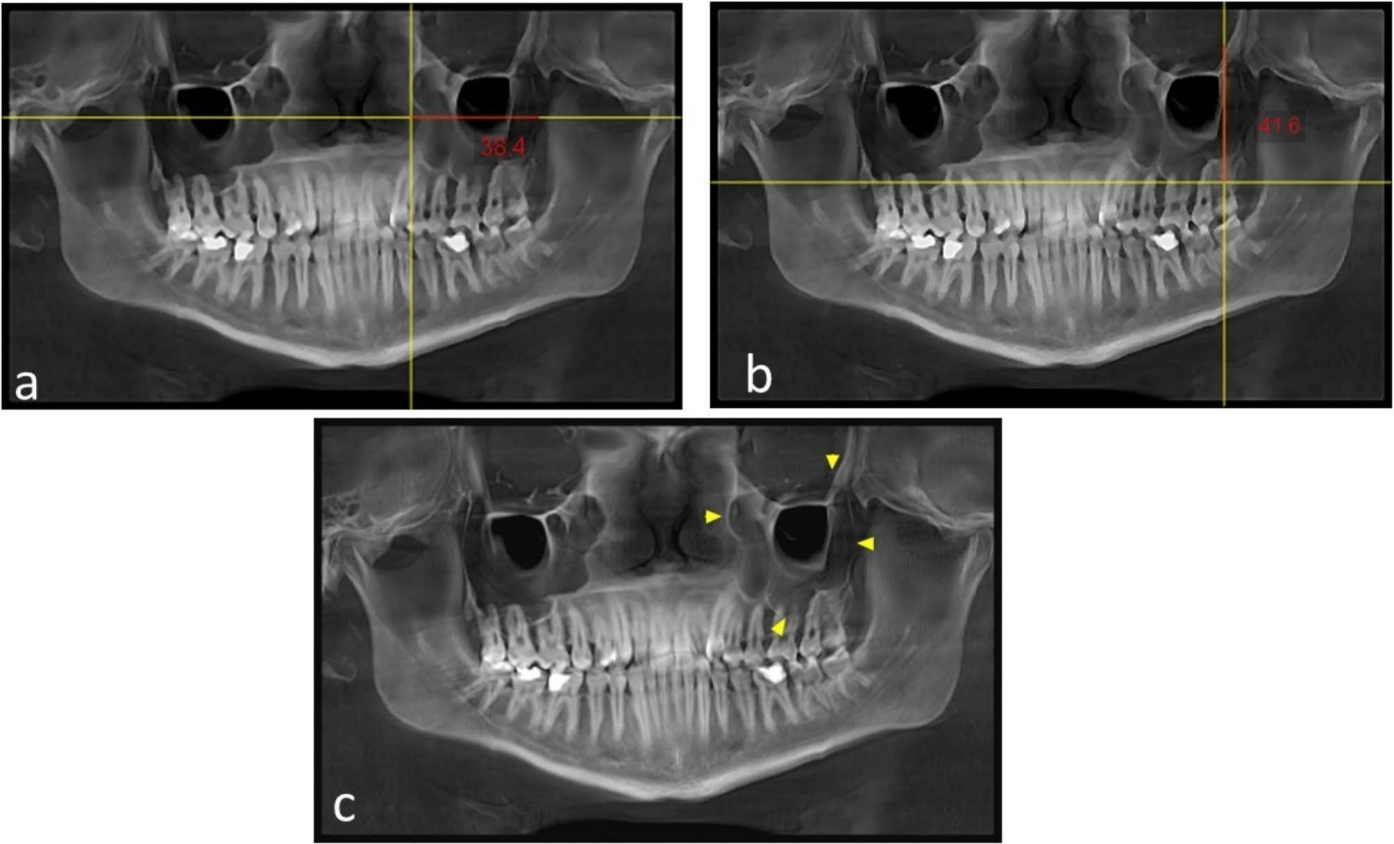
**a** CBCT reformatted panoramic image showing the Max. maxillary sinus width (red line). **b** CBCT reformatted panoramic image showing the Max. maxillary sinus height (red line)
2. Max. maxillary sinus height in mm (Fig. 4b, c).

N.B: Max. maxillary sinus width and height were measured by passing the horizontal and vertical lines available in the software tools through the maximum width and height margins.

3 Max. maxillary sinus hypothetical surface area (the width and height measurements of 182 each sinus were multiplied and resulted in a surface area in mm2).

A randomly selected 25% of the images underwent re-evaluation by both specialists following a one-week interval, from which inter- and intra-observer reliability were computed.

After that we statistically correlated all aforementioned (axial, coronal and sagittal) parameters with the 2D dimension obtained from the reconstructed panoramic image.

#### Statistical analysis

Statistical analysis was performed using SPSS 16 ^®^ (Statistical Package for Scientific Studies), windows excel and Graph pad prism.

All quantitative data were accessible as mean and standard deviation. The study data originated from normal distribution according to the Shapiro-Wilk test and Kolmogorov-Smirnov test. Independent t test was used for comparing all measured parameters among males and females. Measurements of height, width and hypothetical surface area among different GPC pathways in coronal and sagittal planes were performed using One Way ANOVA test followed by Tukey’s Post Hoc test for pairwise comparisons. Regarding Qualitative data, data demonstrated as frequency and percentages, where all comparisons were performed using Chi square test, and Fisher’s Exact test. The results were considered significant when *P* ≤ 0.05. Inter and intra-observer reliability were shown in Table 1, and were measured by Inter Class Correlation coefficient, which showed high to perfect reliability in all measured parameters as (0.945 ≤ ICC ≤ 1).

 Intraclass Correlation Coefficient (ICC), ICC > 0.90 = excellent reliability

**Table 1:** Intra-observer and Inter-observer reliability:

	Interobserver				Intraobserver			
	(ICC)	95% Confidence Interval		*P* value	(ICC)	95% Confidence Interval		*P* value
		Lower Bound	Upper Bound			Lower Bound	Upper Bound	
GPC pathway type/coronal	1.000	1.000	1.000	< 0.0001*	1.000	1.000	1.000	< 0.0001*
GPC pathway type/sagittal	1.000	1.000	1.000	< 0.0001*	1.000	1.000	1.000	< 0.0001*
GPC pathway angle/coronal	0.997	0.992	0.999	< 0.0001*	0.998	0.994	0.999	< 0.0001*
GPC pathway angle/sagittal	0.996	0.991	0.999	< 0.0001*	0.998	0.996	0.999	< 0.0001*
MMS–IF–GPF angle	0.983	0.958	0.993	< 0.0001*	0.998	0.996	0.999	< 0.0001*
GPC axis angle	0.999	0.998	1.000	< 0.0001*	0.999	0.997	1.000	< 0.0001*
GPF-MMS distance (mm)	0.960	0.900	0.984	< 0.0001*	0.988	0.972	0.995	< 0.0001*
GPF(A)−1st molar distance (mm)	0.998	0.996	0.999	< 0.0001*	0.983	0.956	0.993	< 0.0001*
GPF(P)−1st molar distance (mm)	0.983	0.956	0.993	< 0.0001*	0.960	0.900	0.984	< 0.0001*
MS height (mm)	0.978	0.945	0.991	< 0.0001*	0.945	0.867	0.977	< 0.0001*
MS width (mm)	0.967	0.918	0.987	< 0.0001*	0.949	0.876	0.979	< 0.0001*

*GPC *Greater Palatine Canal,* GPF *Greater Palatine Foramen, *MMS* Mid-Maxillary Suture, *IF *Incisive Foramen, *MS* Maxillary Sinus

*Indicates statistically significant agreement (*p* < 0.05)

## Results

Inter and intra-observer reliability were shown in Table 1, which showed high to perfect reliability in all measured parameters as (0.945 ≤ ICC ≤ 1).

1. Description of all the measured parameters among males, females and the overall sample:

Mean and standard deviation of the measured parameters among males, females and the overall sample were presented in Table 2. There was a significant difference between genders regarding GPC pathway angle in the sagittal plane (male patients were significantly lower than female patients; p-value = 0.022); regarding GPF-MMS distance, male patients were significantly higher than female patients (p-value = 0.027); as well as for maxillary sinus dimensions, male patients were significantly higher than female patients (p-value = 0.004, 0.029 and 0.003 for height, width and surface area respectively).

**Table 2:** Comparison of measured parameters between male and female subjects using independent t-test

			Gender						Independent t test		
	Overall		Male		Female				95% Confidence Interval of the Difference		
							Mean Difference	Std. Error Difference			P value
	Mean	Standard Deviation	Mean	Standard Deviation	Mean	Standard Deviation			Lower	Upper	
GPC pathwayangle/coronal	152.27	8.91	152.4	9.41	152.09	8.47	0.35	1.83	3.28	3.98	0.85
Angles											
GPC pathway angle/sagittal	160.30	10.65	157.83	11.83	162.76	8.78	−4.94	2.13	−9.16	−0.71	0.022*
MMS–IF-GPF angle	18.70	3.96	18.56	5.21	18.83	2.12	−0.26	0.81	−1.88	1.35	0.75
GPC axis angle	64.00	8.22	63.81	9.79	64.18	6.38	−0.37	1.69	−3.72	2.97	0.82
Distances											
GPF-MMS distance (mm)	14.74	1.38	15.05	1.43	14.44	1.26	0.62	0.28	0.07	1.16	0.027*
GPF(A)−1st molar distance (mm)	8.23	2.75	8.00	3.03	8.46	2.45	−0.46	0.56	−1.58	0.65	0.41
GPF(P)−1st molar distance (mm)	13.15	2.61	12.95	2.76	13.35	2.47	−0.40	0.53	−1.46	0.66	0.46
GPF(AP) dimension (mm)	4.92	1.52	4.95	1.61	4.88	1.45	0.07	0.31	−0.55	0.69	0.83
MS dimensions											
MS height (mm)	38.83	5.37	40.39	6.12	37.28	3.97	3.12	1.05	1.03	5.21	0.004*
MS width (mm)	38.74	4.23	39.68	4.31	37.81	3.98	1.87	0.85	0.19	3.56	0.029*
MS surface area (mm^2^)	1517.97	335.14	1617.78	367.79	1418.17	267.24	199.61	65.62	69.32	329.9	0.003*

*GPC* Greater Palatine Canal, *GPF* Greater Palatine Foramen, *MMS* Mid-Maxillary Suture, *IF* Incisive Foramen, *MS *Maxillary Sinus, *GPF(A)* Greater Palatine Foramen Anterior Border, *GPF(P)* Greater Palatine Foramen Posterior Border

*Significant difference (*p* ≤ 0.05)

2. Association between gender and GPC pathway in coronal and sagittal planes:

Association between GPC pathways in coronal and sagittal planes and gender was shown in Table 3.

**Table 3:** Distribution of GPC pathway types by gender in coronal and sagittal planes:

		Total		Gender				P value	P value
				Male		Female			
		Count	Column N %	Count	Column N %	Count	Column N %		
GPC path/coronal	A	5	5.2%	5	10.4%	0	0.0%	0.02*	0.01*
	B	87	90.6%	40	83.3%	47	97.9%	0.02*	
	C	4	4.2%	3	6.3%	1	2.1%	0.31	
	D	0	0.0%	0	0.0%	0	0.0%	------	
GPC path/sagittal	a	44	45.8%	16	33.3%	28	58.3%	0.01*	0.01*
	b	26	27.1%	13	27.1%	13	27.1%	----	
	c	26	27.1%	19	39.6%	7	14.6%	0.005*	
	d	0	0.0%	0	0.0%	0	0.0%	-----	
	e	0	0.0%	0	0.0%	0	0.0%	-----	

*Significant difference at *p* ≤ 0.05 (Fisher’s Exact test)

*GPC* Greater Palatine Canal

Regarding the GPC pathway in the coronal plane, most cases showed type B (inferior-lateral from the PPF, then directly inferior) pathway (90.6%). GPC pathway distribution among males and females showed a total significant difference (*p* = 0.01). While most cases of both genders showed GPC pathway of type B (83.3% and 97.9% for males and females, respectively), regarding females, no cases revealed type A (GPC travels directly inferior from the PPF); in contrast, 10.4% of cases in males did (*p* = 0.02); furthermore, type B showed a significant difference between genders (*p* = 0.02). No cases revealed type D pathway among the overall sample.

Regarding the GPC pathway in the sagittal plane, most cases showed type a (an anterior-inferior from PPF) pathway (45.8%). Their distribution among males and females showed a total significant difference (*p* = 0.01): type a was the most prominent GPC pathway among females (58.3%), which showed a significant difference between males and females (*p* = 0.01); in contrast, 39.6% of male cases showed type c (posterior-inferior direction, then an anterior-inferior direction) pathway (*p* = 0.005). No cases revealed type d and e pathway among the overall sample.

3. Correlation between maxillary sinus (MS) height, width, hypothetical surface area and the other measured parameters:

Correlation between MS height, width, hypothetical surface area and the other measured parameters was shown in Table 4.

**Table 4:** Correlation between MS height, width, hypothetical surface area and the other measured parameters:

		MS height (mm)	MS width (mm)	Area
GPC pathway angle/coronal	r	−0.012	−0.063	−0.032
	P value	0.904	0.54	0.757
GPC pathway angle/sagittal	r	−0.408	−0.337	−0.427
	P value	0.0001*	0.001*	0.0001*
MMS–IF–GPF angle	r	−0.054	0.042	−0.018
	P value	0.599	0.682	0.862
GPC axis angle	r	−0.176	−0.109	−0.156
	P value	0.086	0.292	0.128
GPF-MMS distance (mm)	r	−0.08	0.025	−0.035
	P value	0.438	0.811	0.737
GPF(A)−1st molar distance (mm)	r	−0.081	−0.191	−0.167
	P value	0.431	0.062	0.103
GPF(P)−1st molar distance (mm)	r	−0.081	−0.1	−0.115
	P value	0.435	0.33	0.263
GPF(AP) dimension	r	0.008	0.173	0.104
	P value	0.935	0.091	0.312

*GPC* Greater Palatine Canal, *GPF* Greater Palatine Foramen, *MMS* Mid-Maxillary Suture, *IF* Incisive Foramen

*Significant difference as *P* ≤ 0.05

GPC pathway angle in the sagittal plane has shown significant negative correlations with MS height, width and hypothetical surface area (*p* = 0.0001).

4. Association between MS height, width, hypothetical surface area and GPC pathway type in sagittal and coronal plane:

Mean and standard deviation of height, width, and hypothetical surface area among different types of GPC pathway were shown in Table 5.

**Table 5:** Mean and standard deviation of height, width, and hypothetical surface area among different types of GPC pathway:

		GPC pathway/coronal		P value Pairwise	P value		GPC pathway/sagittal		P value Pairwise	P value
		Mean	Standard Deviation				Mean	Standard Deviation		
Hight	A	44.87	5.92	P1 = 0.025* P2 = 0.221 P3 = 0.979	0.03*	a	37.16	5.68	P1 = 0.427 P2 = 0.001* P3 = 0.084	0.002*
	B	38.48	5.02			b	38.73	4.45		
	C	39	8.8			c	41.77	4.51		
Width	A	38.1	3.7	P1 = 0.932 P2 = 1 P3 = 0.955	0.91	a	37.78	3.43	P1 = 0.509 P2 = 0.052 P3 = 0.506	0.06
	B	38.81	4.32			b	38.92	5.03		
	C	38.18	3.41			c	40.21	4.32		
Area	A	1716.74	329.91	P1 = 0.368 P2 = 0.592 P3 = 0.998	0.39	a	1412.59	296.86	P1 = 0.345 P2 = 0.002* P3 = 0.14	0.003*
	B	1507.53	332.98			b	1522.75	345.55		
	C	1496.53	404.5			c	1691.54	323.93		

P1 = pairwise comparison between type A/a and Type B/b, significant difference as *P* ≤ 0.05, P2 = pairwise comparison between type A/a and Type C/c, significant difference as *P* ≤ 0.05, P3= pairwise comparison between type B/b and Type C/c, significant difference as P≤ 0.05

*GPC *Greater Palatine Canal

*Significant difference as *P* ≤ 0.05

Regarding MS height, there was an overall significant difference among GPC pathways in coronal planes (*p* = 0.03), where Type A represented the highest mean height (44.87 mm). There was also a significant difference between Types A and B (p1 = 0.025). In the sagittal plane, there was an overall significant difference in MS height among GPC pathways (*p* = 0.002), where Type c showed the highest mean height (41.77 mm). Furthermore, there was a significant difference between Types a and c (p2 = 0.001).

Regarding MS hypothetical surface area, there was an overall significant difference among GPC pathways in sagittal planes (*p* = 0.003), where Type c showed the highest mean surface area (1691.54 mm²). Furthermore, there was a significant difference between Types a and c (p2 = 0.002). Width measurement of MS showed no significant difference among GPC pathways in both coronal and sagittal planes (*p* > 0.05).

## Discussion

The current study employed the maxillary sinus, a primary anatomy visible on panoramic radiographs, for estimating the variant greater palatine canal pathways and related anatomy that must be examined in CBCT images.

### GPC pathways in coronal and sagittal planes

Regarding GPC pathway in coronal plane, Multiple studies, including Howard-Swirzinski et al. 2010 [4], Sheikhi M et al. 2013 [7], and the current investigation, consistently identified an “inferior-lateral followed by direct inferior” route as the most common GPC pathway in the coronal plane, using the reconstructed CBCT images. Making use of CT scans; Tomaszewska IM et al. 2010 [15] presented slightly more complex, coronal pathway beginning inferiorly, then inferior-lateral, and finally inferior-medial. There is a strong harmony across studies (Howard-Swirzinski et al. 2010 [4], Tomaszewska IM et al. 2010 [15], and the current investigation) that an “anterior-inferior” direction is a predominant GPC’s pathway in the sagittal plane. Sheikhi M et al. 2013 [7] discovered that the GPC moves anteriorly inferiorly after first traveling inferiorly.

Dentists and maxillofacial surgeons can employ the knowledge about the specific GPC pathway to provide critical landmarks for needle insertion, needed for optimum anesthetic efficacy, safer surgical intervention procedures involving the GPC surrounding anatomical structures.

The current investigations showed that there was a significant difference between genders regarding the GPC pathway. In sagittal images, GPC type c pathway (posterior-inferior then an anterior-inferior direction) was prominent regarding males (39.6%), while type a (anterior-inferior direction) was the domain pathway in females. In coronal images, regarding males; 10.4% of cases showed a direct inferior pathway (type A) with no comparable cases in females.

The current study gender-specific variations regarding the GPC pathway, can enhance nerve block techniques. As males tend to have more complex pathway"posterior-inferior then an anterior-inferior direction"in the sagittal plane, profound anesthesia might need to account for this longer, curved pathway compared to the more direct anterior-inferior path common in females.

### GPC pathway angle in coronal and sagittal planes

Hwang SH, et al. 2011 [2] assessed PPF and GPC using 3D-reconstructed CT scans, while Bahşi İ, et al. 2019 [9] evaluated these structures using CBCT sagittal images. The mean GPC route angle was 150.03° ± 9.27° and 159.8° ± 7.1°, respectively, with no discernible gender differences.

The current study showed that the GPC pathway angle was 152.27° ± 8.91° in coronal planes and 160.30° ± 10.65° in sagittal planes, which was close to the aforementioned studies results. In contrast to Hwang SH, et al. 2011 [2] and Bahşi İ, et al. 2019 [9]; the current study results showed a significant difference between males and females regarding GPC pathway angle in sagittal planes (*p* = 0.022), which could be due to employing a different population.

### GPF-MMS distance

Kim DW, et al. 2023 [16] reported the GPF-MMS distance across 38 studies was 15.22 mm in a systematic review and meta‑analysis. In addition, Hwang SH, et al. 2011 [2] explained a significant gender difference regarding the GPF-MMS distance, with a mean distance of 16.2 ± 1.3 mm.

In contrast to Ortug A and Uzel M, 2019 [17], who showed no discernible gender differences in the GPF-MMS distance, measuring 14.64 ± 2.20 on the right side and 14.74 ± 2.22 on the left. Bahşi İ, et al. 2019 [9] observed a significant gender difference in the GPF-MMS distance measured at the axial and coronal planes (14.98 ± 1.45 mm and 13.92 ± 0.86 mm, respectively, *p* = 0.001).

According to the current study’s results using coronal CBCT images, the GPF-MMS mean distance indicates that females’ Medio-Lateral dimensions are comparatively lower (14.74 ± 1.38 mm, *p* = 0.027), which is consistent with Shalaby S, et al. 2015 [18]. This study which analyzed dried Egyptian skulls, also found that the gender differences were significant (*p* < 0.05), with the entire GPF-MMS mean distance for the right and left sides being 14.25 ± 1.7 and 14.17 ± 1.6, respectively. These similarities could give a potential regional anatomical trait.

The consistent mean GPF- MMS distance across numerous studies provide a reliable anatomical benchmark for a variety of dental and surgical procedures including local anesthesia, orthognathic surgery, implantology and palatal soft tissue grafts. Regarding females, as exhibit a shorter GPF-MMS distance, caution approach should be taken during the aforementioned procedures to avoid the damaging of neurovascular bundle.

### MMS–IF–GPF angle

Tomaszewska IM, et al. 2014 [19] examined the MMS–IF–GPF angle on adult, dried human skulls and CT-reconstructed images. They found that the mean angle for males and females was 25.6° ± 2.9° and 26.5° ± 2.9°, respectively, with a significant difference between them (*p* < 0.0001). Gibelli D, et al. 2017 [20] also reported results close to that of Tomaszewska IM, et al. 2014 [19], where they discovered a significant gender difference (*p* < 0.05), and mean MMS–IF–GPF angles of 23.6° ± 2.1° for the right side and 24.2° ± 2.4° for the left.

Shalaby S, et al. 2015 [18] found that the mean MMS–IF–GPF angle on the right and left sides of all Egyptian skulls was 22.9° ± 4.4° and 23.2° ± 4.1°, respectively. This is somewhat different from the current study’s findings (18.56° ± 5.21° and 18.83° ± 2.12° for males and females, respectively). Both Shalaby S, et al. 2015 [18] and the current study results showed no significant difference between males and females. The mean values and the presence or absence of gender related differences may be due to population-specific variations, and potentially age-related factors, as the location of GPF may vary depending on the ossification centers between the maxilla and the palate.

### The angle between GPF and the horizontal plane

Methathrathip D, et al. 2005 [21], Hwang SH, et al. 2011 [2] and Shalaby S, et al. 2015 [18] all reported no discernible difference between the sexes in the angle between GPF and the horizontal plane of the hard palate (57.9° ± 5.8°, 67.4° ± 6.9°, and 40.48° ± 9.1°, respectively).

Bahşi İ, et al. 2019 [9], evaluated the angle between the GPF and horizontal plane using a methodology mimicked the current study, also concluded that there was no discernible gender difference. Their mean angle for the right and left sides was 65.94° ± 6.79° and 65.16° ± 6.10°, respectively. The current study’s mean angle between the GPC axis and the horizontal plane was 64° ± 8.22°, *p* > 0.05, which aligns with the previously cited study results [9]. 

Unlike some other craniofacial dimensions, the angle between GPF and the horizontal plane seems to be a sex-independent feature, which is reflected by the strong consistency across multiple studies, making the clinical negotiation of the posterior maxilla in procedures like GPC nerve block, and planning of implant placement easier.

### GPF-molars distance

Tomaszewska IM, et al. 2014 [19] evaluated the distance between the center of GPF and the center of 2nd and 3rd molars. They reported that, the mean distance to 2nd molar was 12.2 ± 2.3 and 11.4 ± 2 for males and females, respectively. For the 3rd molar, the mean distance was 11.5 ± 2.2 and 11 ± 1.9 for males and females, respectively, with a strong significant difference between genders (*p* < 0.0001).

Regarding the current study, we measured the distance between the distal surface of 1 st molar and both the anterior and posterior borders of GPF; as 1 st molar is the first permanent molar to erupt in the oral cavity. While 1 st molar-GPF anterior border mean distance was 8.23 ± 2.75, 1 st molar-GPF posterior border mean distance was 13.15 ± 2.6. There was no significant difference between genders regarding GPF-1st molar distance.

The inconsistency in results and gender significance could be due to using different reference points for measurements. In the current study, using the 1 st molar as a landmark for various surgical procedures considering the GPC could be highly practical for clinicians, especially in younger patients or those with missing posterior teeth.

### GPF AP dimension

Based on the systematic review made by Kim DW, et al. 2023 [16], the pooled mean of GPF AP dimension across 11 studies was 5.34 mm. While Hwang SH, et al. 2011 [2] reported GPF AP mean dimension of 4.5 ± 0.7 mm, with no significant gender different, Tomaszewska IM, et al. 2010 [15], however, showed a significant different between genders regarding GPF AP measurement (5.1 ± 0.4 for males versus 5 ± 0.4 for females, *p* < 0.0001). Bahşi İ, et al. 2019 [9] reported a higher value for GPF AP diameter (6.44 mm for females and 6.64 mm for males, with no significant different).

Similar to Shalaby S, et al. 2015 [18] who employed an Egyptian population and reported a mean AP dimension of 4.86 ± 0.9 for GPF, with no significant different between genders, the current study showed a GPF AP dimension of 4.95 ± 1.61 and 4.88 ± 1.45 for males and females respectively with no significant different between genders. The similarities between the current investigations and Shalaby S, et al. 2015 [18] results could suggest a potential regional consistency in GPF AP dimensions. GPF AP dimension could influence the needle depth and path during GPC nerve block.

### MS dimensions

As traditional panoramic radiographs and CBCT are equally accurate in identifying the anatomical boundaries of the MS [22], the current investigations used CBCT-generated panoramic images for MS evaluation, and dimension measurement instead of the 2D panoramic radiographs.

De Queiroz CL, et al. 2016 [23] found that males had greater mean values for both MS height and width than females. De Queiroz CL, et al. 2016 [23] found that the mean height and width of the MS regarding females was 28.78 ± 3.39 mm and 44.61 ± 4.62 mm, 27.71 ± 3.92 mm and 45.18 ± 2.83 mm, respectively for left and right MS. The mean height and width of males MS was 30.99 ± 3.38 mm and 48.77 ± 4.24 mm, 30.74 ± 3.59 mm and 48.57 ± 4.49 mm, respectively for left and right MS.

Divyadharsini V and Maheswari TNU, 2023 [24] assessed the height and width of the MS and found that regarding females it was 25.32 ± 3.39 mm and 42.4 ± 4.26 mm, 24.67 ± 4.26 mm and 43.25 ± 2.83 mm, respectively for left and right MS. The mean height and width of males MS was 29.32 ± 3.43 mm and 47.54 ± 3.42 mm, 28.68 ± 4.38 mm and 45.37 ± 4.24 mm, respectively for left and right MS. The aforementioned study showed that males had a greater MS height than females, with no significant difference between right and left sides of the same individual.

Like De Queiroz CL, et al. 2016 [23], the current study showed that males had greater mean values of MS height, width and hypothetical surface area than females (40.39 ± 6.12 mm, 39.68 ± 4.31 mm and 1617.78 ± 367.79mm2, respectively for males in contrast to 37.28 ± 3.97 mm, 37.81 ± 3.98 mm and 1418.17 ± 267.24mm2 for females, *P* ≤ 0.05), where height values were greater than width values.

Understanding the typical height and width of the maxillary sinus, along with their sexual dimorphism, is paramount for planning sinus lift and implant placement procedures. In the current study, the anatomical observation of being the height values greater than width values, may reflects a unique population characteristic.

### Correlation of the MS dimensions to GPC pathway and angle

Medio-posterior position of GPC to the maxillary sinus [25] and the high percent of MS pneumatization posterior to it [26], consolidate the aim of the current study to correlate the MS dimensions to GPC pathway.

The current study results showed that the greater the GPC angle in the sagittal plane, the lesser the MS height, width and hypothetical surface area will be. The aforementioned results coincide with that regarding females who have the lesser MS height, width and surface area, GPC Type a (strait forward anterior-inferior pathway) was the domain one, while in males Type c (a posterior-inferior direction, then an anterior-inferior pathway) shown the highest mean height among all GPC pathways.

In coronal planes, Type A (canal descending directly inferior) represented the greatest mean height, while in sagittal plane, Type c showed the highest mean height and surface area. MS Width showed no obvious effect upon the GPC pathway in both coronal and sagittal planes.

Corelating the MS 2D dimensions to different GPC pathways in sagittal and coronal planes conclude that MS of greater height, has more posterior extension towards the GPC, and MS height dimension had the upper hand on estimating that posterior extension and its possible effect upon GPC angle and pathway.

Due to the posterior relation of the Great Palatine Canal to the maxillary sinus, if the maxillary sinus expands in a posterior direction, it could potentially narrow the GPC pathway and impinge upon the space occupied by the pterygopalatine fossa. This, in turn, could affect the course of the GPC. In the current study, males, who had overall greater MS dimensions, showed GPC Type c (a posterior-inferior direction, then an anterior-inferior pathway) in CBCT sagittal images, with lesser GPC angle, which could reflect the posterior extension of the maxillary sinus dimension and the more susceptibility of MS posterior pneumatization in males than females.

The limitations of this study the respect to population characteristics, and sex difference, caution should be exercised in procedures dealing with the posterior maxilla, particularly when there is a significant MS posterior extension. Sinus lift procedures, especially those utilizing a palatal approach technique, could risk injuring the GPC neurovascular bundle, which enters the oral cavity in an anterior- inferior direction. Further studies with larger sample size in different populations will be required Different imaging modalities will improve understanding anatomical relationship of greater palatine canal (GPC) pathway and maxillary sinus dimensions. Further studies will be required for definitive and accurate measurements of maxillary sinus in axial, coronal and sagittal cuts to detect the exact dimensions of maxillary sinus and compared it with this current study to detect at what distance can the clinician be away from GPC pathway during surgical procedures of harvesting free gingival graft (FGG) or connective tissue graft (CTG) and also during sinus lifting procedures.

## Conclusions

In conclusion, The present study demonstrated a noteworthy correlation between the dimensions of the maxillary sinus (MS) and the greater palatine canal (GPC) structure, particularly the GPC angle and pathway. There was a sex-based correlation between GPC and larger MS (particularly height), with more posterior route and less angle. Due to possible impingement from MS expansion, these findings have significant clinical implications for reducing the risk of GPC neurovascular bundle iatrogenic damage during posterior maxilla operations, specifically sinus lifting procedures for implant placement. Further studies across larger, more diverse populations, using different imaging modalities if applicable, will improve our understanding of this unique anatomical relationship, improve patient safety, and optimize surgical outcomes, even though the results of the current study may offer a useful insight regarding these anatomical parameters.

## Data Availability

The data that support the findings of this study are available from the corresponding author upon reasonable request.
